# High-density EEG of auditory steady-state responses during stimulation of basal forebrain parvalbumin neurons

**DOI:** 10.1038/s41597-020-00621-z

**Published:** 2020-09-08

**Authors:** Eunjin Hwang, Hio-Been Han, Jung Young Kim, Jee Hyun Choi

**Affiliations:** 1 Center for Neuroscience, Korea Institute of Science and Technology (KIST), 5 Hwarang-ro 14-gil, Seongbuk-gu, Seoul 02792 Republic of Korea; 2 Lablup, Inc., 34 Seolleung-ro, Gangnam-gu, Seoul 06132 Republic of Korea; 3 Program of Brain and Cognitive Engineering, Korea Advanced Institute of Science and Technology, Daejeon, 34141 Republic of Korea; 4 Department of Physics, Kyung Hee University, Seoul, 02447 Republic of Korea; 5 Department of Neural Sciences, University of Science and Technology, 217, Gajeong-ro, Yuseong-gu, Daejeon 34113 South Korea

**Keywords:** Attention, Cortex, Diagnostic markers

## Abstract

We present high-density EEG datasets of auditory steady-state responses (ASSRs) recorded from the cortex of freely moving mice with or without optogenetic stimulation of basal forebrain parvalbumin (BF-PV) neurons, known as a subcortical hub circuit for the global workspace. The dataset of ASSRs without BF-PV stimulation (dataset 1) contains raw 36-channel EEG epochs of ASSRs elicited by 10, 20, 30, 40, and 50 Hz click trains and time stamps of stimulations. The dataset of ASSRs with BF-PV stimulation (dataset 2) contains raw 36-channel EEG epochs of 40-Hz ASSRs during BF-PV stimulation with latencies of 0, 6.25, 12.5, and 18.75 ms and time stamps of stimulations. We provide the datasets and step-by-step tutorial analysis scripts written in Python, allowing for descriptions of the event-related potentials, spectrograms, and the topography of power. We complement this experimental dataset with simulation results using a time-dependent perturbation on coupled oscillators. This publicly available dataset will be beneficial to the experimental and computational neuroscientists.

## Abstract (summary)

**Table:** 

Measurement(s)	functional brain measurement • response to auditory stimulus
Technology Type(s)	electroencephalography (EEG)
Factor Type(s)	stimulation frequency • optogenetic stimulation of basal forebrain parvalbumin neurons
Sample Characteristic - Organism	Mus musculus

Machine-accessible metadata file describing the reported data: 10.6084/m9.figshare.12758720

## Background & Summary

Auditory steady-state responses (ASSRs) are electrophysiological responses evoked by periodically repeated acoustic stimuli, and they are widely used in research in neuropsychiatric and brain-computer interface applications. One of the key factors influencing ASSRs is the stimulation rate, where the magnitude of the ASSRs are large in the gamma-frequency band peaking at 40 Hz^1^. The gamma-band ASSRs, utilized as an index of gamma activity, have been proposed as a biomarker of neuropsychiatric disorders, specifically when a disruption of gamma oscillation could underlie putative deficiency in cortico-cortical communication^2^. Nevertheless, the link between the ASSRs and cognitive function is not clear. While automated bottom-up processing is reflected in the brainstem^3^ and primary auditory cortex^4,5^, pathological or state-dependent ASSRs are mostly observed in the frontocentral electrodes^6–8^. Moreover, active or salient (i.e., top-down) listening leads to more faithful ASSRs^8–10^, but numerous counterexamples (e.g., reduced ASSRs for informative cues^11^, the absence of a medication effect on ASSRs^12–14^) make the results on attentional modulation of ASSRs inconclusive. The failure to identify significant factors for ASSR variables may be due to the limitations of the experimentally testable system or the complexity in human psychiatric conditions. Therefore, ASSR studies in more controllable systems (e.g., animal models) are critical for a fundamental understanding of neural circuitry and the processing of ASSRs, particularly by disassociating the top-down modulation from the stimulus-driven responses in a circuitwise manner.

So far, the ASSR recordings in rodents^15,16^ have demonstrated that the general properties of ASSRs are shared in humans and rodents. This cross-species nature has suggested the ASSRs as a translational tool for the evaluation of psychiatric drugs or disease models^16,17^. Indeed, the application of ketamine has enhanced the gamma-band ASSRs in rats^17^, as seen in human ASSRs^18^. Additionally, the impaired 40-Hz ASSRs in schizophrenia patients^12,14^ has been reproduced in schizophrenia rodent models^19,20^. Nonetheless, the peak at slightly higher stimulation rates in mice^16^ and 20-Hz impairment in schizophrenia rodent models^17,21^ remain discrepancies between humans and rodents, indicating the need for the dissection of common and species-specific natures. Additionally, while the origin of the 40-Hz specific nature is reasonably speculated to be the local gamma oscillations entrained by fast-spiking parvalbumin neurons^22,23^, the global synchrony of ASSRs needs to be understood at the mesoscopic and macroscopic levels. Recently, optogenetic inhibition of particular neurons during ASSRs has been attempted in both bottom-up sensory processing (e.g., parvalbumin neurons in thalamic reticular nuclei^24^) and top-down modulation regions (e.g., parvalbumin neurons in the basal forebrain, BF-PV neurons^25^), presenting the neuronal elements of ASSR modulation. In addition, the cortical topography of ASSRs during optogenetic stimulation of BF-PV neurons has demonstrated the importance of the activity timing of certain neurons in ASSR modulation^26^. These attempts suggest that optogenetic silencing of neural components will contribute to defining the neural circuitry of ASSRs, and millisecond-scale perturbations will elucidate the brainwise signal delivery and integration process of ASSRs.

Here, we provide open access to the high-density EEG dataset of ASSRs collected in unrestrained mice with or without optogenetic stimulation of BF-PV neurons. The unperturbed ASSR dataset is provided for various stimulation rates (10, 20, 30, 40, 50 Hz) of sound pulse trains. The perturbed ASSR dataset is provided for various latencies of optogenetic stimulation (0, 6.25, 12.5, 18.75 ms) during 40-Hz sound stimuli. A 40-channel, 7 μm-thick polyimide-based microelectrode resembling a pinnate leaf records high-density EEG from the mouse skull^27,28^. For the ease of data handling, we discarded the posterior channels and provide 36-channel EEG. The first dataset of unperturbed ASSRs can be used as a reference set of mouse ASSRs in studies evaluating psychiatric drugs or phenotyping the electrophysiology of mutants or in comparative neuroimaging of humans and mice. The second dataset of perturbed ASSRs elucidates the hub role of BF-PV neurons in the cortical gamma oscillation network, which can be a canonical representation of the global workspace^29^. The expected derivatives of perturbed ASSRs are system properties from the perspective of information theory (e.g., communication structure, entropy, reliability and robustness) altered by time-dependent perturbation of BF-PV neurons. We demonstrated its usage by constructing a weakly coupled oscillator model and simulating the influence of time-dependent perturbation on ASSRs in the model, which can be extended to a more realistic model synthetizing bottom-up sensory process and top-down modulation of ASSRs with the accumulation of knowledge on the neural components and process of the ASSRs.

## Methods

### Animals

Six male B6 PV-Cre mice (B6;129P2-Pvalbtm1(cre)Arbr/J, Stock #008069, The Jackson Laboratory, Bar Harbor, ME, USA), aged >10 weeks and weighted >25 g at the surgery and >13 weeks at the recording, were used. Mice were housed on a 12:12 light-dark cycle (lights on at 8 AM) in a room temperature (21 °C) with ad libitum access to food and water. All the experimental procedures were approved by the Institutional Animal Care and Use Committee of the Korea Institute of Science and Technology (Permit number: 2014–027) and were performed according to the guidelines of the Korean Animal and Plant Quarantine Agency (Publication no. 12512, partial amendment 2014) as well as United States National Institute of Health guidelines (NIH publication no. 86–23, revised 1985). No adverse events were observed.

### Stereotaxic surgery

**Fig. 1:**
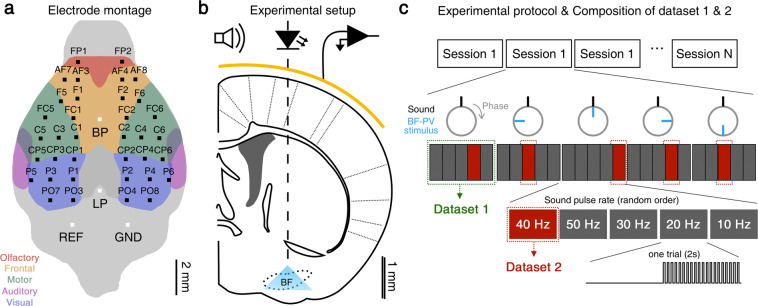
Experimental methods. (**a**) A montage of the electrode array and channel labels. BP: Bregma point, LP: Lambda point. (**b**) Optic stimulation to BF (basal forebrain) and EEG acquisition using electrode array (yellow line). (**c**) Schematic diagram of the experimental protocol and conditions, and composition of dataset 1 & 2.

Virus injection and the implantation of the EEG microelectrode and optic cannula were performed together on a stereotaxic apparatus (Model 900, David Kopf Instruments, Tujunga, CA, USA). First, the mouse was anesthetized with an intraperitoneal injection of a ketamine-xylazine cocktail (120 and 6 mg/kg, respectively) and then fixed on the stereotaxic device. Second, one microliter of adeno-associated viral vector expressing channelrhodopsin2 (AAV5-DIO-EF1a-hChR2[H134R]-EYFP, University of North Carolina Vector Core, NC, USA) was injected into the intermediate part of the left BF (AP, 0.0 mm; ML, 1.6 mm; DV, 5.5 mm from bregma). The target site was determined based on the density of cortically projecting parvalbumin-positive neurons, where it was found to be the highest^30^, and the transfection of the virus makes the parvalbumin-positive neurons excitable by impinging 470-nm light. Third, we inserted a fiber-optic cannula (FT200EMT, Thorlabs Inc., NJ, USA) with a 1.25 mm ceramic ferrule (230 µm inner diameter, Precision Fiber Products Inc., CA, USA) into the same site. Last, we placed a microarray for a high-density EEG probe on the skull (electrode montage in Fig. 1a) and sealed the cannula and microelectrode with dental cement (Vertex Self-Curing, Vertex Dental, Zeist, Netherlands), as depicted in Fig. 1b. After the surgery, antibiotic ointment (Fucidine, Donghwa, Seoul, South Korea) was applied to the incision. More detailed information for the custom-developed microelectrodes and EEG surgery is provided in our previous works^27,28^, and the microelectrode is available upon request to the corresponding author. ChR2 expression was confirmed via immunohistochemistry in the postmortem brain. The data were discarded if the expression of ChR2 and PV cell was not colocalized or if the tip position was not in the basal forebrain.

### Experimental paradigm

The experimental paradigm was illustrated in Fig. 1c. As depicted, one session was composed of six blocks with different BF-PV stimulation conditions, and each block has five epochs with different stimulation frequencies. We tested various BF-PV stimulation conditions: no stimulation (i.e., sound only), continuous stimulation, pulse stimulation with different phase delays of 0, π/2, π, and 3π/2, referred as *in-phase*, *delayed*, *out-of-phase*, and *advanced* stimulation, respectively. We tested 5 different sound pulse rates (i.e., stimulation frequency, *f*_*s*_) ranging from 10 to 50 Hz with 10 Hz interval, and they were given in a random order. The time delay is the phase delay divided by 2πfs. For example, at *f*_*s*_ = 40 Hz, the tested phase delays are 0, 6.25, 12.5, and 18.75 ms with respect to sound onset. Each epoch is composed of 1 s of stimulation and 2 s of interstimulation interval. The stimulation is composed of 1-ms sound pulses with or without 1-ms LED light pulses (470 nm, 1.2 mW, M470F1, Thorlabs Inc., Newton, NJ, USA). The stimuli were given to a mouse placed in a 500-mL glass beaker by impinging a cylindrical symmetrical sound way with four speakers (85 dB at the center, Britz International, Paju, South Korea). The sound and light pulse trains were driven separately through a DAQ analog output module (#9263, National Instruments, Austin, TX, USA) and an LED driver (DC2100, Thorlabs Inc., NJ, USA), respectively. More detailed information on the experimental paradigm is provided in our previous work^26^. Within all blocks, we sorted sound-only epochs into Dataset 1 and 40-Hz epochs with time-dependent stimulation of BF-PV neurons into Dataset 2. Dataset 2 has been analyzed and used to be published in our previous work^26^.

### High-density EEG recording and analysis

The EEG recording was performed during the light phase in a custom-made soundproof Faraday cage. The high-density EEG was sampled at 2 kHz using a SynAmps2 amplifier and a SCAN 4.5 data acquisition system (Neuroscan Inc., El Paso, TX, USA) and bandpass filtered between 0.1 and 100 Hz. The electrode-skull contact impedances in most channels were < 500 kΩ. Two channels above the interparietal bones were used as reference and ground electrodes. Single-trial epochs were extracted from −1 to 2 s relative to sound stimulus onset and sorted for the same stimulation conditions in terms of sound rates and optogenetic stimulation latencies. The data were detrended by subtracting the mean amplitude of each epoch, and the epochs containing artifacts were removed. The event-related potentials (ERPs) were obtained by averaging the time series of all epochs. The event-related spectral power was obtained by

1 $$power(f,t)=\left(\frac{1}{{N}_{epoch}},\mathop{\sum }\limits_{n=1}^{{N}_{epoch}},{|{F}_{n}(f,t)|}^{2},-,{\mu }_{B},(,f,)\right)/{\sigma }_{B}(f),$$

where *F*_*n*_ is the spectral estimate of n-th epoch computed using FFT, *N*_*epoch*_ is the number of epochs (Tables 1 and 2), and *μ*_*B*_ and *σ*_*B*_ are the mean and standard deviation of the baseline spectral power, respectively.

**Table 1:** Number of trial epochs included in dataset 1.

Animal	Stimulation condition					Total
	10 Hz	20 Hz	30 Hz	40 Hz	50 Hz	
1	95	95	98	84	94	466
2	98	95	97	87	98	475
3	96	95	97	53	93	434
4	98	96	95	72	98	459
5	191	191	189	176	189	936
6	196	194	195	179	194	958
Total	774	766	771	651	766	3,728

**Table 2:** Number of trial epochs included in dataset 2.

Animal	Stimulation condition					Total
	*Sound-only*	*Advanced*	*In-phase*	*Out-of-phase*	*Delayed*	
1	84	84	79	88	82	417
2	87	79	74	77	83	400
3	53	56	45	51	50	255
4	72	76	71	70	72	361
5	176	86	167	96	89	614
6	179	86	179	77	86	607
Total	651	467	615	459	462	2,654

Note that the data of the ‘Sound-only’ condition are equal to those of the ‘40 Hz’ condition in dataset 1.

## Data Records

Data files were submitted to the GIN server of G-Node (REF: 10.12751/g-node.e5tyek), containing a total of 5,731 epochs (see Tables 1 and 2 for details) from 6 animals (*Mus musculus*). The dataset can be accessed by the GIN website directly (10.12751/g-node.e5tyek)^31^, by the *gin* command-line tool (gin get hiobeen/Mouse hdEEG ASSR Hwang et al), or by the custom-written Python function (download_dataset()) included in the dataset (Demo 1-1 of *analysis_tutorial.ipynb* or *download_sample.py*). A copy of dataset is also available in Zenodo server^32^ (10.5281/zenodo.3949519).

The GIN repository contains three types of data: (1) csv-formatted tables, *meta.csv* containing demographic information of each subject and *montage.csv* containing channel coordinate information of electrode array; (2) raw data files in EEGLAB format, *datasetN/epochs_animalN.fdt* and*.set* containing EEG data of animal ID; and (3) a step-by-step tutorial document written in IPython-Notebook, *analysis_tutorial.ipynb*. To open the raw data files after download, the installation of the EEGLAB toolbox (in the MATLAB environment) or the MNE-Python toolbox (in the Python environment) is required. The tutorial covers basic file-handling operations such as downloading to conventional EEG analyses (see Technical Validation for details), such as event-related potential analysis, time-frequency analysis, and power topography. The tutorial document is fully functioning with the Google Colaboratory environment, and it is highly recommended to look through it online before getting started on your local machine.

## Technical Validation

The high-density EEG dataset of ASSRs in mice was validated by producing the topographical representation of the event-related spectral power for different sound repetition rates. The high-density EEG dataset of ASSRs under time-dependent perturbation of BF-PV neurons was validated by producing the time-frequency analysis of ASSRs and the topographical representation of gamma power for different BF-PV latencies. Here, we used animal ID #2. Note that all the figures presented here are generated via Python scripts available in the tutorial, and the same analysis of other animals can be easily reproduced by making a few changes in the tutorial document.

### ASSRs in mice

**Fig. 2:**
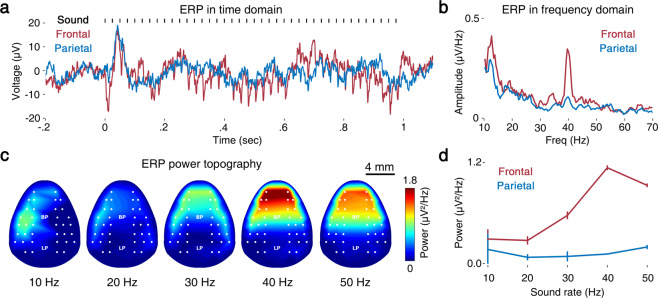
Event-related potentials and topography analysis of ASSRs from a sample subject (Animal No. 2 in Tables 1 and 2). (**a**) Trial-averaged response to the sound stimulus (black) in the frontal (red) and parietal (blue) areas and (**b**) its frequency-domain representation. (**c**) Power topography of each condition. (**d**) Mean ASSR power of frontal (channel 3 to 6) and parietal (channel 21 to 24) channels.

The ASSR literature in human EEG has commonly presented ERPs followed by sustained activity during the period of sound^11^ and the maximal ASSRs in the vertex and/or middle frontal area^6–8^. Figure 2a illustrates the transient evoked potentials for the frontal and parietal cortex, successfully reproducing ASSRs in human EEG^11^. The signals in the frontal (channels = AF03, AF04, AF07 and AF08) and parietal cortex (channels = CP01, CP02, CP03, and CP04) were averaged to produce the local ERPs. While ERPs were elicited in both cortices, the sound-locked responses in the frontal cortex were more manifest than those observed in the parietal cortex. The comparison of the mean power spectra for the frontal and parietal cortex is shown in Fig. 2b. The topographic maps were obtained by averaging over stimulation time and epochs at each channel (Fig. 2c). Except for low-frequency stimulation (<20 Hz), the ASSRs were strongest in the frontal cortex, as in the human ASSRs^6–8^. The strength increases as the sound rate increases, which is summarized in the sound rate-response curves (Fig. 2d). The sound rate-dependent nature of the ASSRs observed in humans was successfully reproduced in the mouse frontal ASSRs by delivering similar topographical representations^7^ and rate-response curves^12^.

### Modulation of the ASSRs by BF-PV latency

**Fig. 3:**
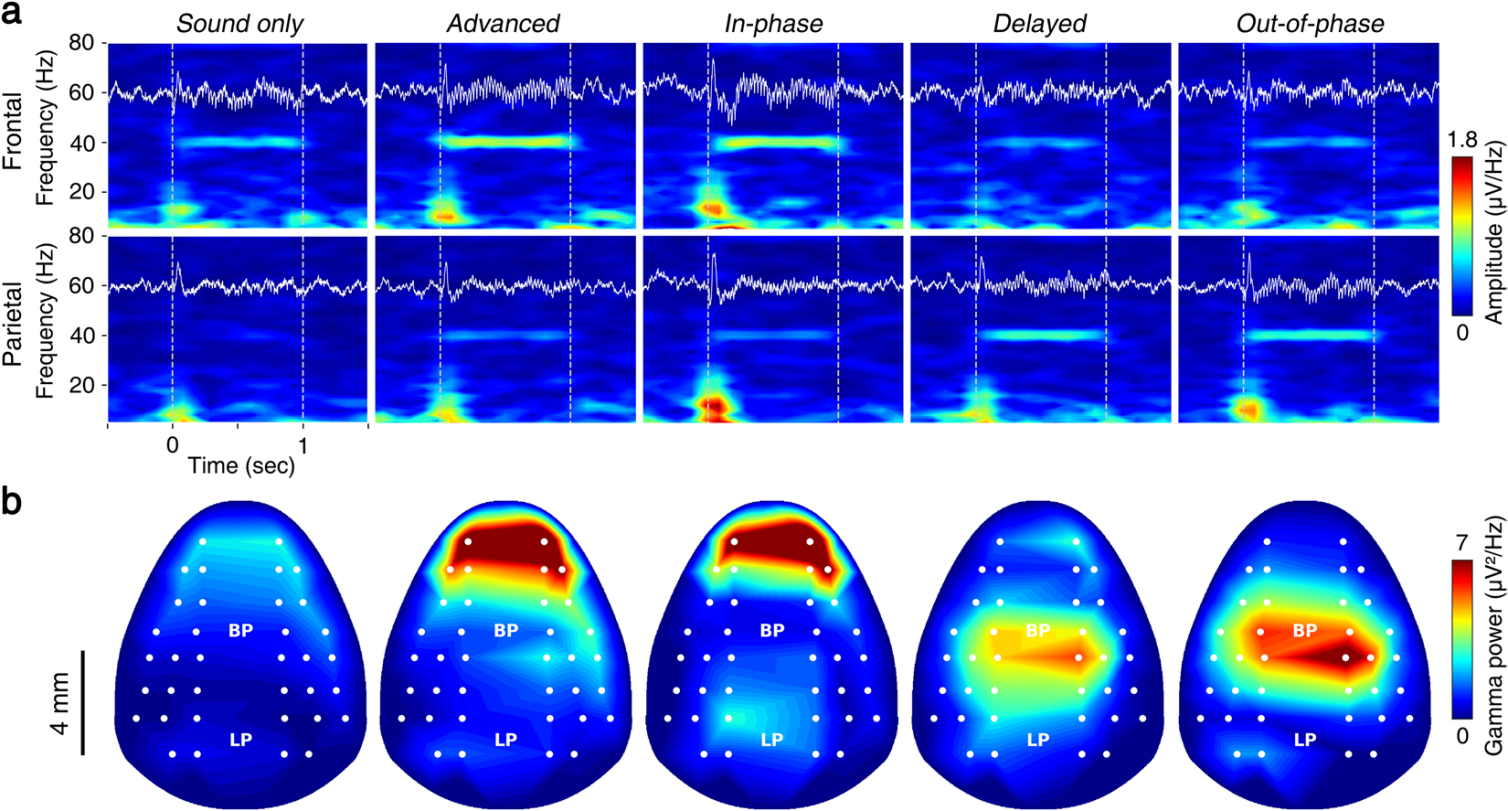
Cortical gamma-ASSR and its optogenetic perturbation from a sample subject (Animal No. 2 in Tables 1 and 2). (**a**) Time-frequency representation of ERPs for various experimental conditions. Note that the white solid line indicates the time trace of ERPs, and white dashed lines indicate the onset and offset of auditory stimulation. (**b**) Power topography of the gamma-frequency band (38–42 Hz).

BF-PV neurons are known to modulate cortical gamma activities, and the inhibition of BF-PV neurons significantly reduces the ASSRs^25^. We compared the 40-Hz ASSRs with or without BF-PV stimulation and investigated the modulation effect of BF-PV latency. The grand average time-frequency maps of event-related spectral power present the BF-PV latency-dependent behaviors of the ASSRs (Fig. 3a). The frontal ASSRs were modulated by BF-PV latency in a constructive way for *advanced* and *in-phase* activation but in a destructive way for *delayed* and *out-of-phase* activation. It is noteworthy that the parietal ASSRs emerged in the destructive condition for the frontal ASSRs. The topographies of gamma power manifest these case-sensitive effects in the space domain (Fig. 3b). The enhanced ASSRs during *advanced* or *in-phase* stimulation of BF-PV neurons mimics the attentional modulation of ASSRs observed in humans^8,10^, whereas the impaired ASSRs during *out-of-phase* or *delayed* stimulation of BF-PV neurons resembles the deficits of ASSRs observed in psychiatric patients^12,14^.

### Simulation of the roles of BF-PV neurons in the emergence of the ASSR network

**Fig. 4:**
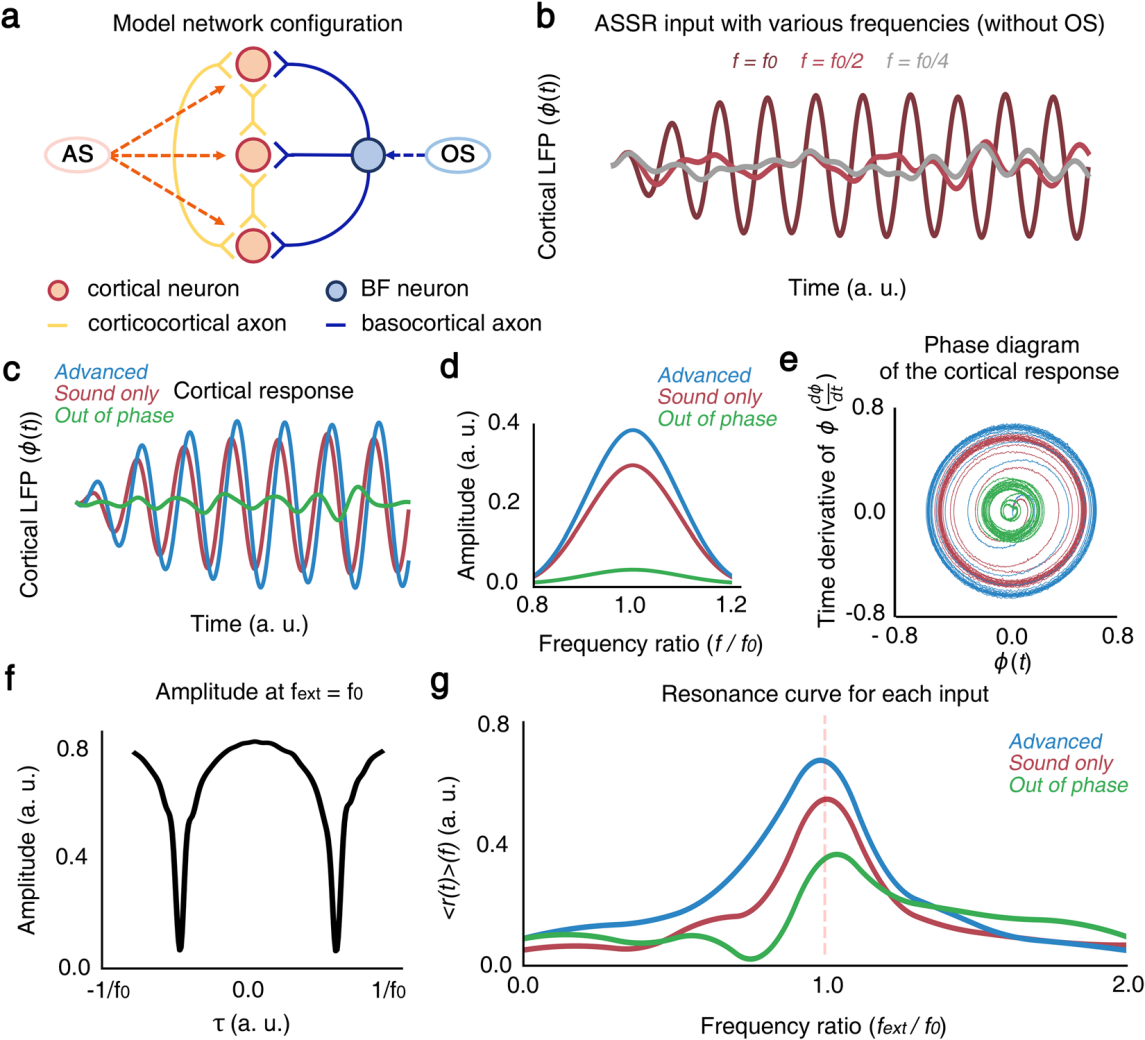
Results for the model equation for each experimental condition. (**a**) A simple diagram of this model. AS indicates auditory stimulation, OS indicates optogenetic stimulation. AS gives stimulation with some Gaussian noise but with appropriate OS; auditory stimulation’s SNR becomes larger since BF neurons cancel out the noise of the auditory stimulation. (**b**) Amplitude of LFP, *ϕ*(*t*) simulated for various stimulation frequencies, *f* = *f*_*0*_, *f*_*0*_/2, and *f*_*0*_/4, colored by brown, red, and green, respectively. *f*_*0*_ is the intrinsic frequency of the network. (**c**) Amplitude of LFP, *ϕ*(*t*) simulated for auditory stimulation with or without optogenetic intervention according to (Eq. 6). The LFP evoked by auditory stimulation (red) was elevated by *advanced* optogenetic stimulation (blue), whereas *out-of-phase* intervention abolished the evoked LFP (green). Here, the phase delays were -π/2 and π for *advanced* and *out-of-phase* optogenetic stimulations, corresponding to −1/(2 *f*_*0*_) and 1/*f*_*0*_ in time. (**d**) The power spectrum of LFP. (**e**) The trajectories of LFP in a phase diagram of *ϕ*(*t*) and dϕ(*t*)/d*t*. (**f**) The delay response curves of LFP for time-dependent optogenetic stimulation during ongoing auditory stimulation, where the frequency of auditory and optogenetic stimulation, *f*_*ext*_ was same as the intrinsic frequency, *f*_*0*_. (**g**) The frequency response curves of LFP with respect to various stimulation frequencies, *f*_*ext*_.

BF-PV neurons are known to increase cortical activation during sensory stimulation^33^ and generate cortical gamma oscillations^25^. The second dataset presents the timing effects of BF-PV intervention on the ongoing ASSR rhythms. The BF-PV intervention produced unique combined effects leading to synergetic effects produced by the whole cortex in terms of network structure and system robustness^26^. While the resonance property^23^ of PV interneurons in a frequency domain explains the local gamma oscillations, the nonlinear effect of BF-PV neurons on the global ASSRs suggests its role as a subcortical hub for top-down control of ASSRs. Here, we validate this phenomenon by deriving a forced Kuramoto model with time-varying parameters^34^, as depicted in Fig. 4a.

2 $$\frac{d{\theta }_{i}}{dt}={\omega }_{i}+\frac{1}{N}\mathop{\sum }\limits_{j}^{N}K\left({\theta }_{ij}\right)\sin \left({\theta }_{i}-{\theta }_{j}\right)+{{\rm{F}}}_{{\rm{AS}}}\left({\theta }_{i},\,t\right)+{{\rm{F}}}_{{\rm{OS}}}\left({\theta }_{i},\,t\right),$$

for $i=1,...,N$. Here, *θ*_*i*_ is the phase of the i^th^ oscillator, *ω*_*i*_ is its natural frequency of the i^th^ oscillator, N is the number of oscillators, and *K*(*θ*_*ij*_) is the coupling strength with resonance property at *f*_0_ as

3 $$K\left({\theta }_{ij}\right)=\frac{{k}_{max}}{\sqrt{{\left({\left({\dot{\theta }}_{i}\right)}^{2}-{\left(2\pi {f}_{0}\right)}^{2}\right)}^{2}+1}\sqrt{{\left({\left({\dot{\theta }}_{j}\right)}^{2}-{\left(2\pi {f}_{0}\right)}^{2}\right)}^{2}+1}},$$

where *k*_*max*_ is the maximum value of *K*(*θ*_*ij*_).

For computational efficiency, we set *f*_0_ = 0.2 Hz and defined the external stimulus function (i.e., auditory stimuli with Gaussian noise), F_AS_ (*θ*, *t*), as

4 $${{\rm{F}}}_{{\rm{AS}}}\left(\theta ,\,t\right)={A}_{AS}\sin \left(2\pi {f}_{AS}t+\xi \left(\mu ,\,{\sigma }^{2}\right)-\theta \right),$$

where A_*AS*_ and *f*_*AS*_ are the amplitude and frequency of the auditory stimuli and $\xi \left(\mu ,\,{\sigma }^{2}\right)$ is the Gaussian noise with the expectation value *μ* and the variance *σ*^2^. Next, we defined the optogenetic input, F_OS_ (*θ*, *t*), as

5 $${{\rm{F}}}_{{\rm{OS}}}\left(\theta ,\,t\right)={A}_{OS}\sin \left(2\pi {f}_{OS}\left(t+{\tau }_{lag}+\tau \right)-\theta \right),$$

where A_*OS*_ and *f*_*OS*_ are the amplitude and frequency of the optogenetic stimuli, *τ*_*lag*_ is the synaptic time delay from the BF to the cortex and τ denotes the latency of optogenetic stimulation with respect to sound onset. All parameter values used in the model are described in Table 3. By combining (Eqs. 2–5), Eq. (2) becomes

6 $$\begin{array}{llll}\frac{d{\theta }_{i}}{dt} & = & {\omega }_{i}+\frac{1}{N}\mathop{\sum }\limits_{j}^{N} & \frac{{k}_{max}}{\sqrt{{\left({\left({\dot{\theta }}_{j}\right)}^{2}-{\omega }_{0}^{2}\right)}^{2}+1}}\sin \left({\theta }_{i}-{\theta }_{j}\right)\\  &  &  & +{A}_{AS}\sin \left(2\pi {f}_{AS}t+\xi \left(\mu ,\,{\sigma }^{2}\right)-{\theta }_{i}\right)\\  &  &  & +{A}_{OS}\sin \left(2\pi {f}_{OS}\left(t+{\tau }_{lag}+\tau \right)-{\theta }_{i}\right),\end{array}$$

for ${\rm{i}}=1,\ldots ,{\rm{N}}$. To describe the synchronization, the order parameter $r\left(t\right),\psi \left(t\right)$ is defined by

7 $${{\rm{re}}}^{i\psi }\equiv \frac{1}{N}\mathop{\sum }\limits_{j=1}^{N}{e}^{i{\theta }_{j}}.$$

here, *r*(*t*) represents the magnitude of the mean field, which represents the degree of synchronization between cortical neurons, and $\psi (t)$ is the phase of the mean field. In this case, $\phi \left(t\right)=r\left(t\right)\sin \psi \left(t\right)$ resembles the LFP signal.

**Table 3:** The parameter values used in the model.

Condition	*N*	*k*_*ma*x_	*f*_0_	A_*AS*_	A_*OS*_	*f*_*AS*_	*f*_*OS*_	*τ*_*lag*_	*τ*
Sound only	1000	0.8	0.2	0.3	·	0.2	·	·	·
Advanced	1000	0.8	0.2	0.3	0.09	0.2	0.2	$\frac{1}{12{f}_{0}}$	$-\frac{{\rm{\pi }}}{2}$
Out of phase	1000	0.8	0.2	0.3	0.09	0.2	0.2	$\frac{1}{12{f}_{0}}$	π

*ω*_i_ was selected from normal distribution with *μ* = 0 and σ = 0.5,

*θ*_*i*_(0) was selected from normal distribution with *μ* = 0 and σ = 20,

(μ: an average of the normal distribution, σ: a standard deviation of the normal distribution.

Figure 4b shows the ASSRs for various sound repetition rates, successfully producing the resonance property of ASSRs at gamma frequencies. The interaction of coupled oscillators led the emergence of synchronized oscillations, whose amplitudes are modulated by sound repetition rates. Figure 4c illustrates the latency effect during time-dependent perturbation of ASSRs, summarized as resonance curves for different perturbation conditions (Fig. 4d). In the *advanced* condition, cortical activities (i.e., EEG) become more synchronized (higher $r$) than *sound only* condition. On the other hand, *r* is lower than *sound only* condition for all time in the *out-of-phase* condition. When optogenetic stimulation applied with various τ, the amplitude at *f*_*OS*_ = *f*_0_ becomes the smallest when τ = 1/2f_0_ corresponds to *out-of-phase* condition (Fig. 4e). Figure 4f shows the limit cycles of forced coupled oscillators whose radius is modulated by perturbation latency. We summarized the simulation results in the latency response curves (Fig. 4g). In sum, the forced Kuramoto model not only provide a unifying framework, but also successfully simulate the behaviors of datasets.

## Usage Notes

The mouse ASSR data acquired by high-density EEG are publicly available in EEGLab^35^ file format in the GIN G-node repository^31^. We present the full lengths of raw time-series as well as the archive of epochs sorted for different conditions and the example codes in Python to produce the time-frequency power plots and topographies in Figs. 2–3. For MATLAB users, the open-source MATLAB software EEGLab^35^ or Fieldtrip^36^ can be used to assist in processing the EEG data. For Python users, MNE toolbox permit to handle *.set files (https://mne.tools/stable/index.html) and for R users, eegUtils package permits to handle *.set files (https://craddm.github.io/eegUtils/index.html). A step-by-step tutorial to deal with mouse EEG is publicly available in the fieldtrip repository (http://www.fieldtriptoolbox.org/tutorial/mouse_eeg/), where the basic EEG analysis such as preprocessing, artifact rejection, ERP analysis, and time-frequency power plots, as well as the estimation of an equivalent dipole source for 3D representation of dipole source-localization, are provided and demonstrated for known sources of optogenetic deep brain stimulation^37^. For group analysis of ASSRs, an analysis demonstration based on FieldTrip can be helpful^38^. The dipole source localization of ASSRs can be used in comparing the datasets obtained from functional magnetic resonance imaging (fMRI) techniques. For the convenience of fMRI researchers, we provided our dataset in BIDS format^31^ as well, a standardized dataset for organizing and describing MRI datasets^39^.

The data sets documented here will allow us to quantify the functional connectivity of the mouse cortex, as indicative of physiological/cognitive states, as exemplified in our previous work (see Fig. 5B-C in Hwang, *et al*.^26^). Such classification can be conducted either on independent condition sets or in data-driven ways. The classified functional connectivity can be used in describing the state of the mice or regarded as surrogates for neurodynamic markers representing any pathological states.

## Data Availability

All the Python scripts used in the *Technical Validation* section for analysis and figure generation are available online^31^. Python scripts for simulation are also available in G-Node repository (*simulation/run_simulation.ipynb*).
